# Bevacizumab-Associated Enterocutaneous Fistula: A Case Report and Management Review

**DOI:** 10.7759/cureus.92925

**Published:** 2025-09-22

**Authors:** Madison L Wallace, Linda Akbarshahi, Matthew Bond, Tembele Yangandawele, Thomas Howdieshell

**Affiliations:** 1 Obstetrics and Gynecology, Medical College of Georgia, Augusta University, Athens, USA; 2 Family Medicine, Northeast Georgia Medical Center Gainesville, Gainesville, USA; 3 Surgery, Northeast Georgia Medical Center Gainsville, Gainesville, USA; 4 Family Medicine, Northeast Georgia Medical Center Gainsville, Gainesville, USA; 5 Trauma Surgery, Medical College of Georgia, Augusta University, Athens, USA

**Keywords:** anti-vegf therapy, bevacizumab therapy, enterocutaneous fistula, fistula management, immunotherapy side effects, locally advanced cervical cancer, neoadjuvant bevacizumab, oncology patient, recurrent cervical cancer, recurring cervical cancer

## Abstract

Enterocutaneous fistula (ECF) formation is a rare but severe complication of bevacizumab therapy. This case report documents the first known patient to develop both an ECF and a colovaginal fistula following bevacizumab treatment for recurrent cervical cancer without prior radiation therapy. A comprehensive literature review of current and nutritional management guidelines, in the context of this patient’s complex condition, highlights the need for refined risk stratification and management frameworks. This report aims to contribute to the evolving understanding of bevacizumab’s adverse effects and inform future therapeutic decision-making.

## Introduction

Bevacizumab is a monoclonal antibody that targets vascular endothelial growth factor (VEGF) to inhibit angiogenesis, which restricts tumor vascularization and growth. Between April 2009 and January 2012, the Gynecologic Oncology Group (GOG) 0240 trial demonstrated that bevacizumab prolonged survival in patients with recurrent or persistent cervical cancer [1]. In October 2021, following the positive outcomes of the GOG 0240 trial, the Food and Drug Administration (FDA) expanded treatment options to include bevacizumab, in conjunction with pembrolizumab and chemotherapy, for patients with recurrent cervical cancer or to treat tumors with positive programmed death-ligand 1 (PD-L1) expression. Even for tumors that lack PD-L1 expression, the current treatment recommendations include chemotherapy with bevacizumab [2]. Both treatment regimens are currently classified as Category 1 recommendations by the National Comprehensive Cancer Network (NCCN) [3].

Although bevacizumab has survival benefits for patients with recurrent cervical cancer, its use is not without significant risk. Potentially life-threatening complications associated with bevacizumab use include delayed wound healing, gastrointestinal (GI) perforation, and fistula formation, which carries a 10% mortality risk [4]. Documented reports of bevacizumab-induced fistulae, which have occurred in at least 6% of patients, have mostly involved rectovaginal, vesicovaginal, and ureterovaginal fistulae in patients with known risk factors, such as prior radiation therapy, extensive surgical history, and tumor invasion [1,5]. Given the increase in bevacizumab use for gynecologic cancers, we must work quickly to modify current risk stratification models to encompass new risk factors and associated complications.

## Case presentation

**Figure 1:**
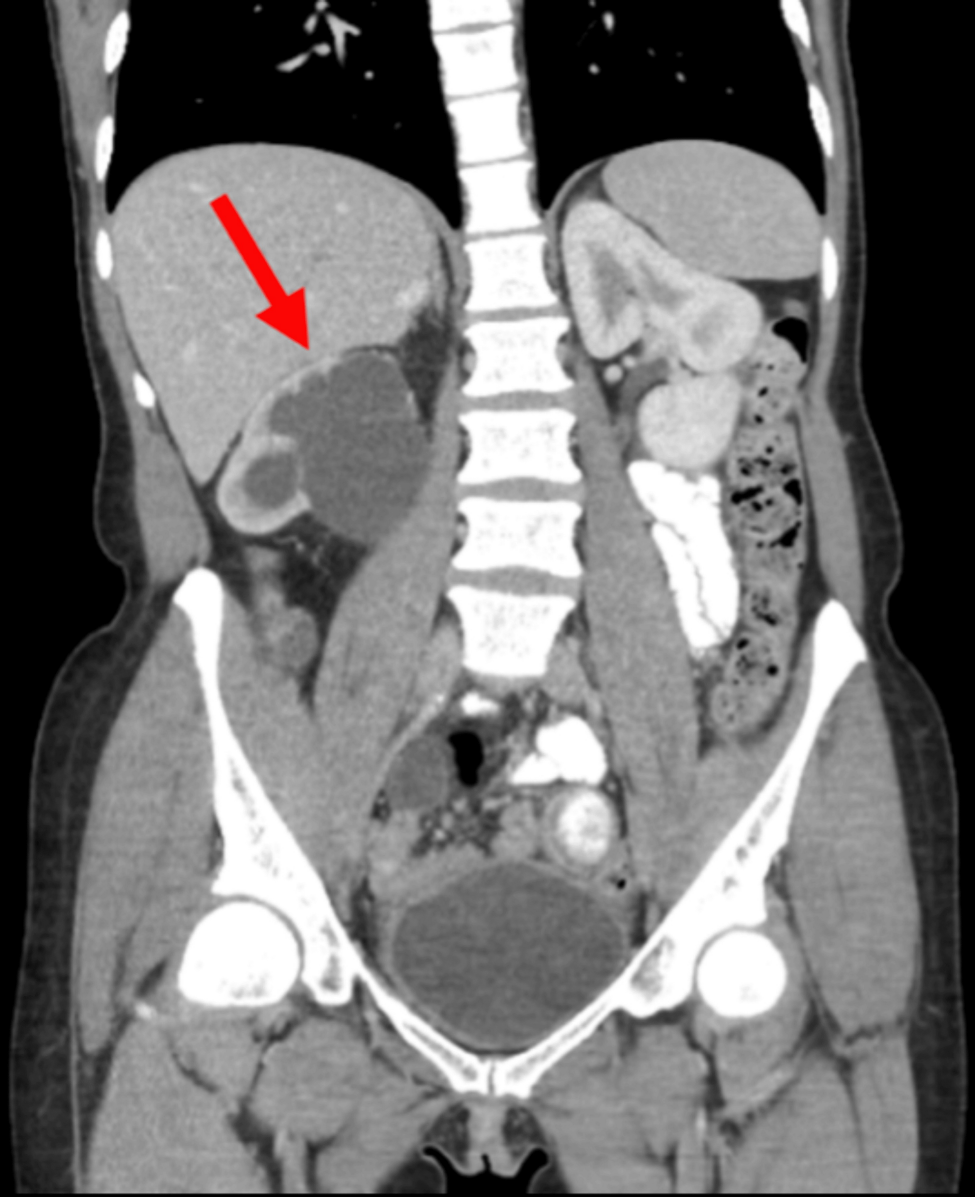
Severe hydronephrosis. The patient underwent PET-CT imaging with injection of 18F-fluorodeoxyglucose, which demonstrated severe hydronephrosis (arrow) of the right kidney. The ureter was diffusely dilated to just beyond the level of the iliac vasculature. The left kidney appeared normal. PET-CT, positron emission tomography-computed tomography

**Figure 2:**
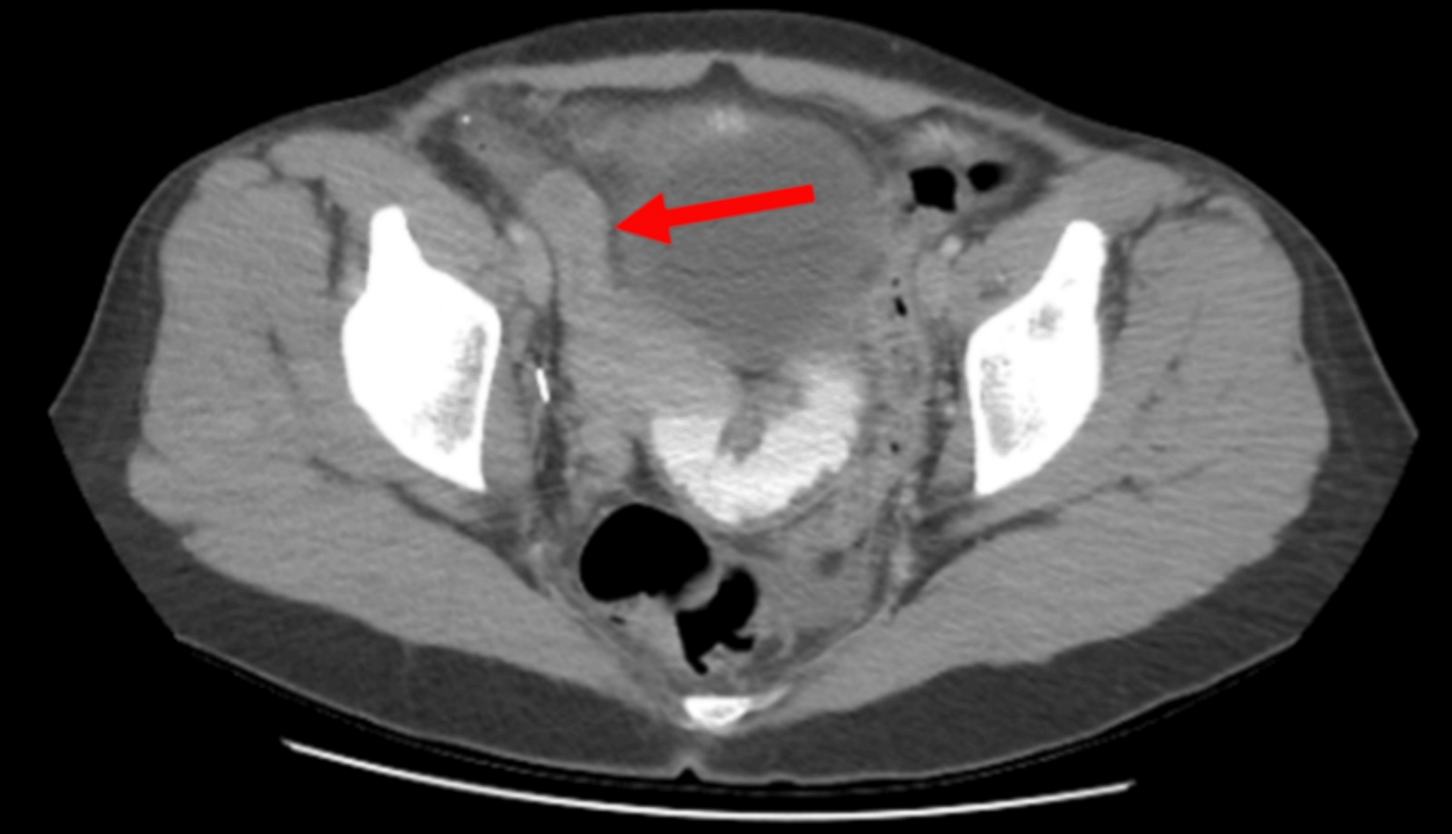
Intramural edema. The patient underwent PET-CT imaging with injection of 18F-fluorodeoxyglucose, which demonstrated wall thickening and intramural edema within the terminal ileum (arrow). The recurrent metastatic soft tissue mass in the right pelvis extends into two segments of the terminal ileum and abuts the right lateral wall of the rectosigmoid junction.

The patient is a woman in her 40s, a current smoker with a 23-pack-year history, previously diagnosed nearly five years ago with Stage IB1, Grade 2 squamous cell carcinoma of the cervix, status post radical hysterectomy, bilateral salpingectomy, bilateral ovarian transposition, and bilateral pelvic lymphadenectomy. Histopathology reports confirmed a moderately to poorly differentiated invasive squamous cell carcinoma, with negative lymph node involvement. Caris molecular testing of the tumor showed positive PD-L1 expression, microsatellite stability, low tumor mutational burden, and negative human epidermal growth factor receptor 2 (HER2)/Neu testing. Six months ago, the patient developed progressive right lower quadrant pain. Positron emission tomography (PET) and computed tomography (CT) imaging identified a 5 x 5 cm infiltrative pelvic mass, determined to be oligometastatic disease within the pelvis, encasing the distal right ureter, causing severe hydronephrosis (Figure 1) and involving two segments of small bowel mural edema without obstruction (Figure 2).

**Figure 3:**
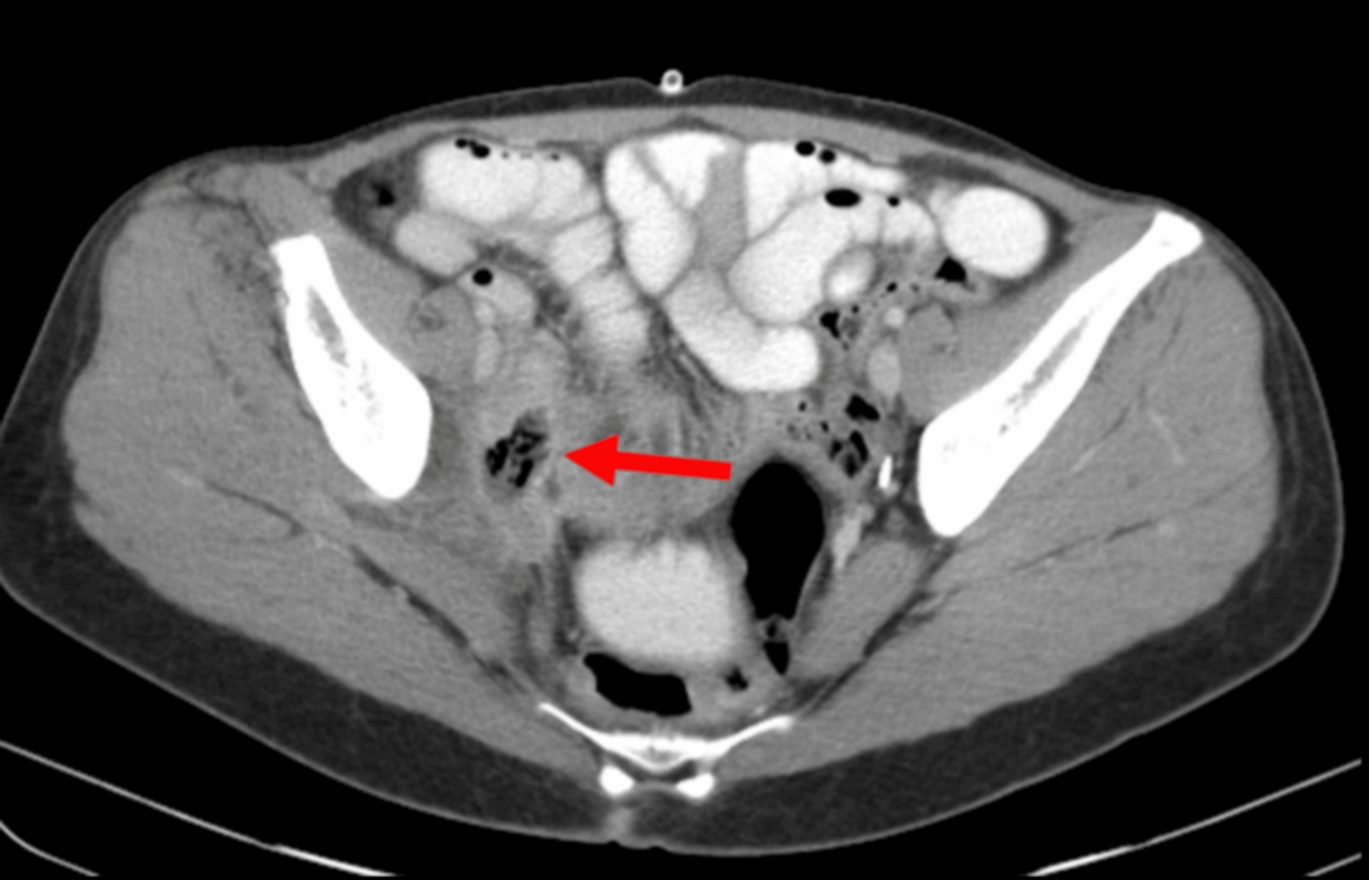
Pelvic collection of gas and fluid. The patient underwent CT imaging of the abdomen and pelvis with oral contrast, which demonstrated a gas and fluid collection along the right pelvic sidewall (arrow) measuring 2 × 3 × 2 cm, immediately adjacent to loops of small bowel and colon and associated with the previously noted abnormality.

To alleviate the ureteral obstruction, she underwent placement of a percutaneous nephrostomy tube. Given the size, location, and positive PD-L1 expression, current guidelines recommended a regimen consisting of pembrolizumab, carboplatin, paclitaxel, and bevacizumab for a total of six treatments before surgical resection of the mass. Her insurance company denied coverage for pembrolizumab, so she only received the other three recommended drugs. Her last treatment cycle was administered one month before her presentation to the hospital. A follow-up CT scan was performed to evaluate for treatment response, and it revealed a gas and fluid collection associated with the previously seen abnormality (Figure 3).

The following month, she presented to the hospital with worsening right lower extremity and abdominal pain, which began after her most recent chemotherapy cycle. On examination, the patient was afebrile but tachycardic. The right lower extremity was erythematous, swollen, and markedly tender to palpation. Her abdomen was visibly distended with diffuse tenderness, most pronounced in the suprapubic region, without rebound or guarding. Initial laboratory evaluation revealed a normocytic anemia (10.6 g/dL), a corrected non-anion gap metabolic acidosis of 10, and hypoalbuminemia (2.8 g/dL), consistent with poor nutritional status (Tables 1-2). She did not meet Systemic Inflammatory Response Syndrome (SIRS) or sepsis criteria on admission due to the absence of fever, leukocytosis, and tachypnea. However, these findings did not diminish our concern for an underlying infectious process, given her recent neoadjuvant chemotherapy course and the overall clinical picture.

**Table 1:** Complete blood count. MCV, mean corpuscular volume; MCH, mean corpuscular hemoglobin; MCHC, mean corpuscular hemoglobin concentration; RDW SD, red cell distribution width - standard deviation; RDW CV, red cell distribution width - coefficient of variation; MPV, mean platelet volume

Component	Value	Flag	Reference range	Units
White blood cells	9.7		4.8-10.8	x10E3/µL
Red blood cells	3.62	Low	4.20-5.40	x10E6/µL
Hemoglobin	10.6	Low	12.0-16.0	g/dL
Hematocrit	32.3	Low	37.0-47.0	%
MCV	89.2		81.0 - 99.0	fL
MCH	29.3		27.0-31.0	pg
MCHC	32.8	Low	33.0-37.0	g/dL
RDW SD	58.4	High	35.1-43.9	fL
RDW CV	18	High	11.5-14.9	%
Platelet count	193		130-400	x10E3/µL
MPV	9.7		9.5-14.4	fL

**Table 2:** Comprehensive metabolic panel. IU, International Units; AST, aspartate aminotransferase; ALT, alanine aminotransferase; EGFR, estimated glomerular filtration rate; CKD-EPI, Chronic Kidney Disease Epidemiology Collaboration

Component	Value	Flag	Reference range	Units
Sodium	134		135-148	mmol/L
Potassium	3.8		3.5-5.2	mmol/L
Chloride	100		100-110	mmol/L
Bicarbonate	27		21-32	mmol/L
Urea Nitrogen	14		5.0-23.0	mg/dL
Creatinine	0.7		0.60-1.00	mg/dL
Glucose	208	High	65-99	mg/dL
Calcium	9		8.4-10.6	mg/dL
Total protein	6.7		6.0-8.3	g/dL
Albumin	2.8	Low	3.4 -5.0	g/dL
Albumin/globulin ratio	0.72	Low	0.90-2.00	
Total bilirubin	0.28		0-1.00	mg/dL
Alkaline phosphatase	122		45-136	IU/L
AST	31		0-48	IU/L
ALT	50		13-61	IU/L
Anion gap	7		4.3-12.3	mmol/L
EGFR (CKD-EPI)	107.2		>=60	mL/min/1.73 m^2^

**Figure 4:**
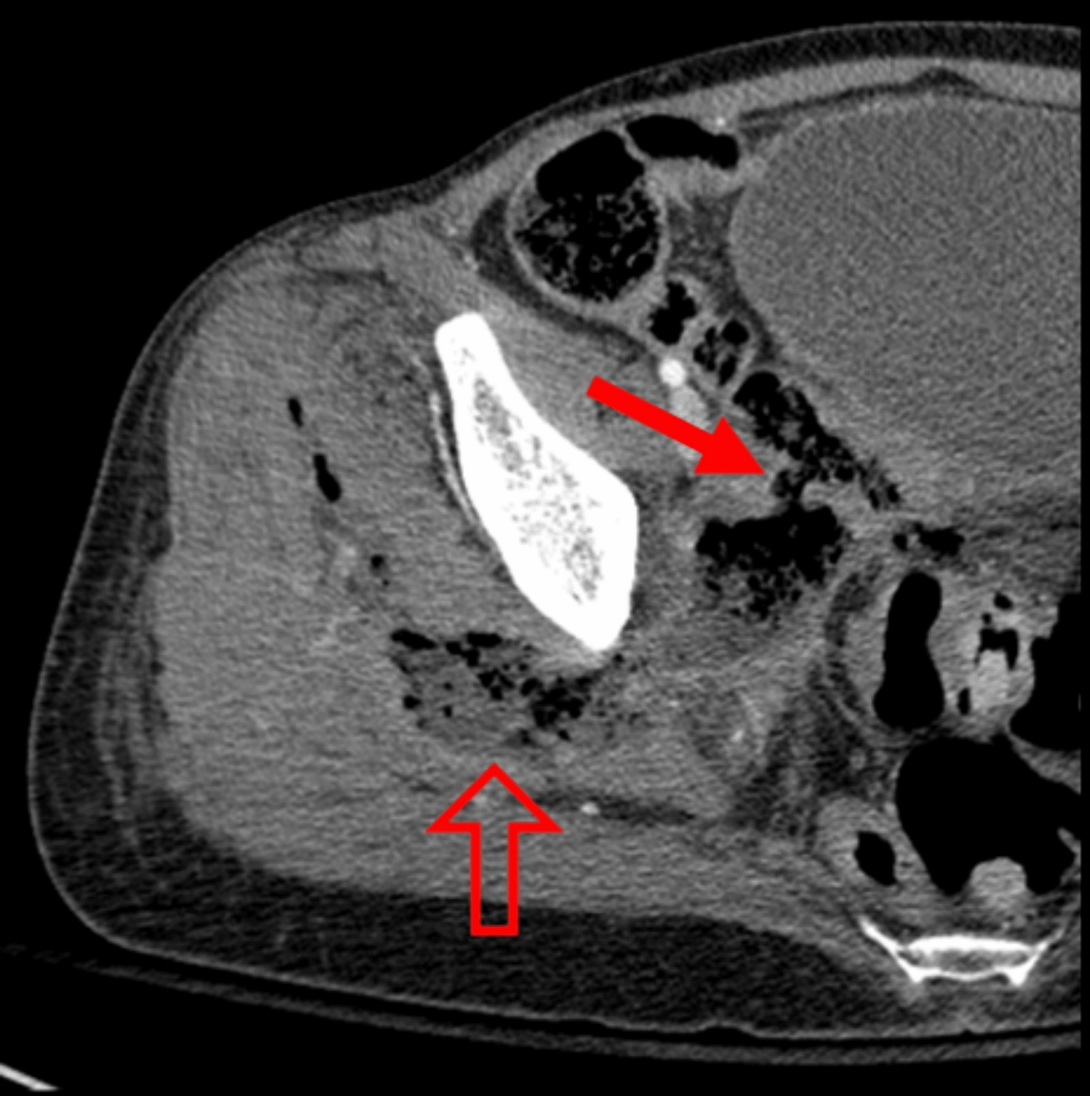
Fistula tract extending from a distal small bowel loop to an intrapelvic fluid collection. The patient underwent CT imaging of the abdomen and pelvis with intravenous (IV) contrast, which demonstrated a complex collection of fluid and gas in the right hemipelvis, extending into the iliopsoas muscle and gluteal musculature (unfilled arrow). A thin tract is visible along the anterior aspect of the intrapelvic fluid collection, extending to and appearing contiguous with a distal small bowel loop located posteromedial to the cecum (arrow).

A CT scan of the abdomen and pelvis with contrast revealed an abscess (Figure 4) in the right lower extremity, which prompted surgical intervention.

**Figure 5:**
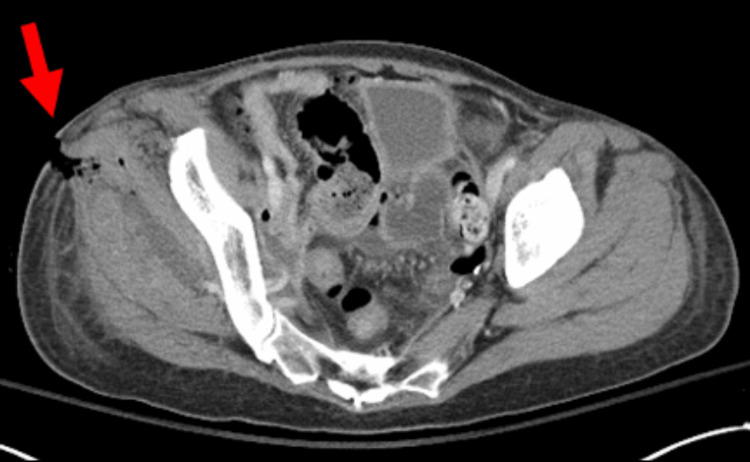
Enterocutaneous fistula. The patient underwent CT imaging of the abdomen and pelvis with oral contrast, which demonstrated an evolving abscess in the right buttocks extending into the pelvis and now in direct communication with the skin (arrow), creating an enterocutaneous fistula.

Incision and drainage (I&D) of the fluid collection soon followed. Multiple purulent pockets of fluid were encountered, extending to the femoral head, without any obvious fistulous communication visualized. Nonviable muscle tissue (4 cm²) surrounding these abscess cavities was removed and cultured. Polymicrobial growth was seen on tissue cultures, which included Escherichia coli and Enterococcus faecium, likely seeded by intestinal flora. Broad-spectrum antibiotics were initiated. A repeat CT scan on post-op day 1 showed an evolving abscess in the right buttock, with increased volume and direct communication with the skin (Figure 5).

**Figure 6:**
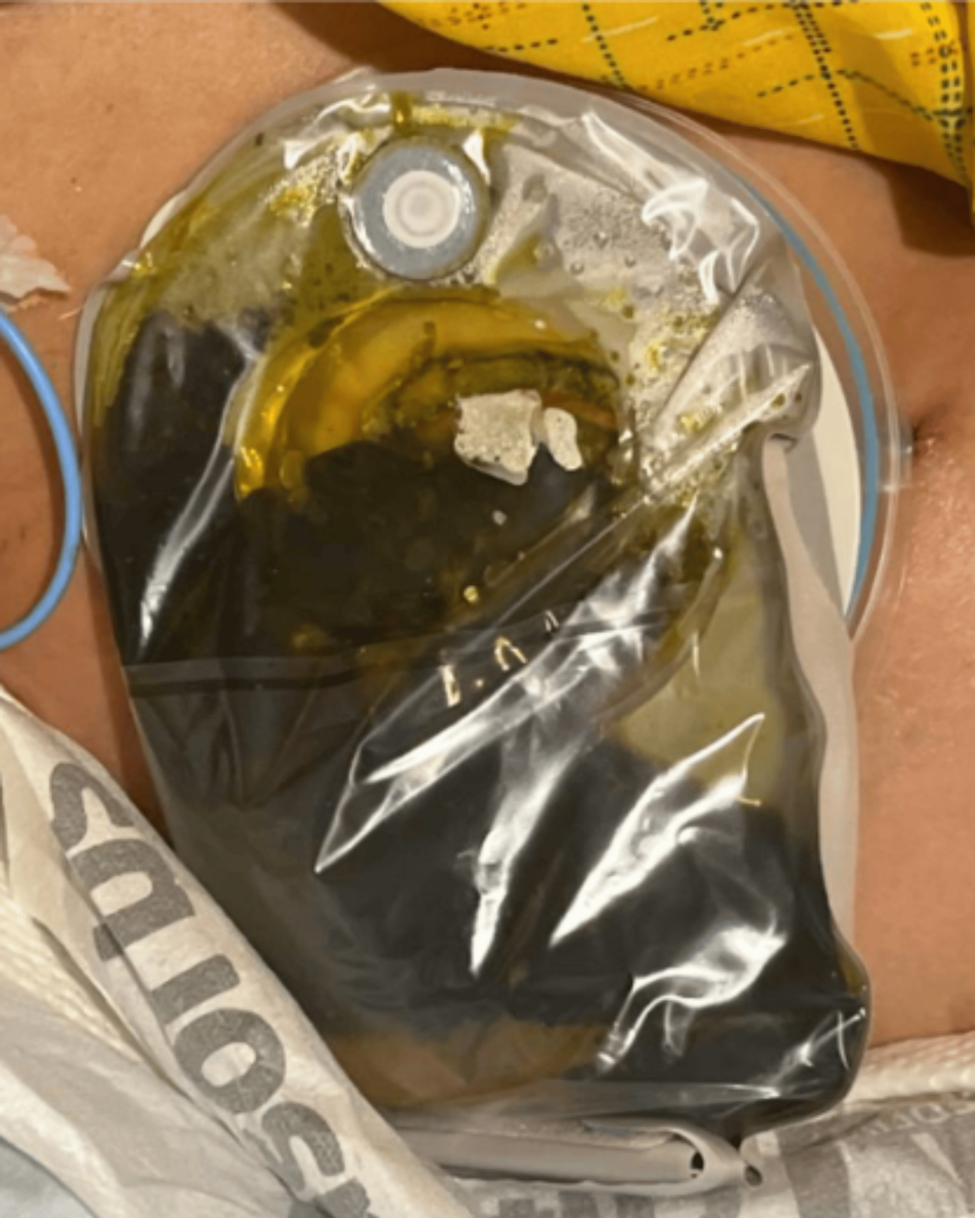
Ostomy bag. Bilious enteric contents were observed exiting from the enterocutaneous fistula on postoperative day 3.

At the bedside, enteric contents were observed draining from the skin incision, necessitating placement of an ostomy bag and daily inpatient wound care (Figure 6). After a week in the hospital, she was discharged on oral antibiotics.

A month later, the patient returned to the hospital for new concerns of fecal matter passing through her vaginal canal with associated abdominal pain. CT imaging revealed the previously seen enterocutaneous fistula (ECF) and associated thigh abscess, with a new possible colovaginal fistula, a second abscess above her bladder, and a small bowel obstruction. IR-guided drainage of the new abscess was performed, and cultures grew Candida albicans. IV micafungin was added on top of the broad-spectrum antibiotics.

All plans for further surgical management of this patient were deferred, given her poor nutritional status and extensive intra-abdominal adhesions. She was placed on total parenteral nutrition (TPN) to optimize nutritional support in preparation for potential future colectomy and definitive surgical repair of both fistulae. The patient was eventually discharged with IV ertapenem and micafungin. Follow-up was arranged with gynecologic oncology, infectious disease, and surgical specialists to monitor for disease progression and assess ongoing fistula and wound complications.

## Discussion

Bevacizumab and the persistent risk of GI complications

Bevacizumab, a monoclonal antibody targeting VEGF, has been instrumental in the therapeutic landscape of recurrent cervical cancer. It has been shown in multiple studies to prolong median overall survival by 17 months (hazard ratio for death from any cause, 0.71; 98% confidence interval (CI), 0.54-0.95) without significantly impacting the patient’s quality of life [2]. Nevertheless, its anti-angiogenic properties compromise tissue repair mechanisms, thereby predisposing patients to GI perforations and fistula formation.

Intestinal perforation occurs in approximately 2%-3% of patients receiving bevacizumab in combination with cytotoxic chemotherapy, prompting the inclusion of a black box warning in the FDA-approved prescribing information [6]. A recent clinical trial compared dose-dense and conventional paclitaxel plus carboplatin, with or without bevacizumab, in metastatic or recurrent cervical cancer treatment. To lessen the risk of GI-related adverse events, bevacizumab was not administered to patients suspected of having GI-involved tumors or intestinal obstruction. Still, GI perforation and fistula formation occurred in 3.7% and 6.1% of patients receiving chemotherapy plus bevacizumab. Life-threatening perforations occurred in two patients with prior pelvic chemoradiotherapy [5].

The GOG 0240 trial documented a markedly increased incidence of complex GI fistulae in patients receiving bevacizumab in conjunction with chemotherapy (cisplatin and paclitaxel) compared to chemotherapy alone (3% vs. 0%), and the frequency of GI fistulae was significantly increased in bevacizumab-containing regimens (6% vs. 0%, *P *= 0.002). However, the trial's documented cases were confined to rectovaginal, vesicovaginal, and ureterovaginal fistulae, with these cases only occurring in patients with prior radiation therapy [1]. It has been previously reported that non-irradiated patients have a 0% fistula risk [7]. Notably, this case represents the first documentation in the literature of ECF formation in recurrent cervical cancer following bevacizumab therapy without radiation therapy, warranting further investigation.

In contrast to bevacizumab, immune checkpoint inhibitors, like pembrolizumab, have shown significant clinical benefits without being linked to fistula formation. The KEYNOTE-158 trial showed a response rate of 14.3% when using pembrolizumab to treat tumors with PD-L1 expression in addition to one or more previous chemotherapy regimens. In the KEYNOTE-826 trial, pembrolizumab plus chemotherapy significantly improved median progression-free survival compared to a placebo plus chemotherapy (10.4 vs. 8.2 months; *P* < 0.001) and overall percent survival at 24 months (53% vs. 41.7%; *P *< 0.001), regardless of bevacizumab use [8]. The ongoing BEATcc trial aims to demonstrate at least a 30% decrease in the hazard of death with a better safety profile when using PD-L1 therapies in addition to the standard recommended therapy of chemotherapy with or without anti-VEGF therapy [9]. Given these findings, it may be safer to prioritize immune checkpoint inhibitors over VEGF inhibitors in the treatment of recurrent cervical cancer. This raises the question of whether the patient might have been able to avoid these complications if she had received pembrolizumab instead of, or in addition to, bevacizumab.

The patient’s history of systemic chemotherapy, prior surgical interventions, and tobacco use most likely increased her risk of developing a fistula, but these factors were not previously studied in clinical trials. Knowing all possible risk factors before initiating therapy is critical when individualizing treatments and educating patients on potential complications. This highlights a critical gap in oncologic decision-making. If bevacizumab can increase the risk of fistula formation, even in the absence of radiation, it is necessary to fully investigate this association to inform future clinical decisions and to optimize current standards for treatment in this vulnerable patient population.

Limitations of standard enterocutaneous fistula management guidelines

While general ECF management strategies have been established and followed in the management of this patient, it is still unclear what the best course of action would be in this unique setting. The American Society for Parenteral and Enteral Nutrition (ASPEN) and the European Society for Clinical Nutrition and Metabolism (ESPEN) advocate a multimodal approach to ECF management, prioritizing hemodynamic stabilization, infection control, nutritional optimization, and timely surgical intervention, which in about 33% of patients can result in spontaneous closure [10,11]. The management of this patient adhered to these principles in several key areas while also underscoring areas necessitating further refinement.

Given the polymicrobial growth on tissue cultures, broad-spectrum antimicrobial therapy was promptly initiated. The patient underwent surgical I&D, mirroring evidence-based infection control protocols [11]. Recognizing the impact of malnutrition and sustained enteric output, the clinical team implemented TPN per ASPEN guidelines and planned to postpone surgical intervention until her albumin rose to >2.2 g/dL. Emerging literature now suggests that enteral nutrition, instead of TPN, may be feasible in select cases, an avenue warranting further exploration for the management of this patient. Recent studies have also shown that serum albumin concentrations can be artificially decreased in the setting of ECF-related inflammation and therefore should not be used as a sole factor in predicting clinical outcomes, though they can have prognostic significance [11,12].

The use of ostomy bags for controlled drainage adhered to best practices in preventing peristomal skin complications and secondary infections, but in this patient without a clear fistulous tract or stoma, an adequate seal and prevention of leakage were not achieved, highlighting an opportunity for protocol improvement. Although negative pressure wound therapy (NPWT) was not documented in this case, its utility in complex fistula disease warrants further consideration [13-17]. Inconclusive and often contradicting outcomes have been reported in ECF outcomes when using NPWT, with the worst outcomes including additional fistula formation and the best outcomes including complete fistula closure in 15 days [13,14].

Expanding our understanding of wound healing and prognosis in the context of anti-angiogenic therapy could help to refine management strategies and improve overall care. It is important to keep in mind that, given the rarity of this GI issue, the quality of evidence underpinning many of these recommendations remains suboptimal [12].

## Conclusions

Bevacizumab use can increase the risk of fistula formation, regardless of prior radiation exposure. Successful management of bevacizumab-associated fistulae requires multidisciplinary collaboration among oncology, infectious disease, surgical, and nutritional specialists. Nutritional optimization is crucial to improving surgical outcomes in enterocutaneous fistula management. Current predictive tools for bevacizumab-related complications lack specificity, necessitating further research into patient selection criteria. Investigating minimally invasive approaches and machine-learning-driven risk models may enhance management and reduce morbidity in this vulnerable patient population.
